# Perivascular spaces as a marker of disease severity and neurodegeneration in patients with behavioral variant frontotemporal dementia

**DOI:** 10.3389/fnins.2022.1003522

**Published:** 2022-10-20

**Authors:** Jasmine Moses, Benjamin Sinclair, Daniel L. Schwartz, Lisa C. Silbert, Terence J. O’Brien, Meng Law, Lucy Vivash

**Affiliations:** ^1^Department of Neuroscience, Central Clinical School, Monash University, Melbourne, VIC, Australia; ^2^Department of Neurology, Alfred Hospital, Melbourne, VIC, Australia; ^3^Department of Medicine, Royal Melbourne Hospital, University of Melbourne, Melbourne, VIC, Australia; ^4^NIA-Layton Oregon Aging and Alzheimer’s Disease Research Center, Oregon Health & Science University, Portland, OR, United States; ^5^Advanced Imaging Research Center, Oregon Health and Science University, Portland, OR, United States; ^6^Department of Neurology, Portland Veterans Affairs Health Care System, Portland, OR, United States; ^7^Department of Neurology, Royal Melbourne Hospital, University of Melbourne, Melbourne, VIC, Australia; ^8^Department of Radiology, Alfred Health, Melbourne, VIC, Australia; ^9^Department of Electrical and Computer Systems Engineering, Monash University, Melbourne, VIC, Australia

**Keywords:** Behavioural Variant Frontotemporal Dementia, frontotemporal lobar degeneration, perivascular spaces, tau, clinical trial, anti-tau therapy, sodium selenate

## Abstract

**Background:**

Behavioural Variant Frontotemporal Dementia (bvFTD) is a rapidly progressing neurodegenerative proteinopathy. Perivascular spaces (PVS) form a part of the brain’s glymphatic clearance system. When enlarged due to poor glymphatic clearance of toxic proteins, PVS become larger and more conspicuous on MRI. Therefore, enlarged PVS may be a useful biomarker of disease severity and progression in neurodegenerative proteinopathies such as bvFTD. This study aimed to determine the utility of PVS as a biomarker of disease progression in patients with bvFTD.

**Materials and methods:**

Serial baseline and week 52 MRIs acquired from ten patients with bvFTD prospectively recruited and followed in a Phase 1b open label trial of sodium selenate for bvFTD were used in this study. An automated algorithm quantified PVS on MRI, which was visually inspected and validated by a member of the study team. The number and volume of PVS were extracted and mixed models used to assess the relationship between PVS burden and other measures of disease (cognition, carer burden scale, protein biomarkers). Additional exploratory analysis investigated PVS burden in patients who appeared to not progress over the 12 months of selenate treatment (i.e., “non-progressors”).

**Results:**

Overall, PVS cluster number (ß = −3.27, CI [−7.80 – 1.27], *p* = 0.267) and PVS volume (ß = −36.8, CI [−84.9 – 11.3], *p* = 0.171) did not change over the paired MRI scans 12 months apart. There was association between cognition total composite scores and the PVS burden (PVS cluster ß = −0.802e^–3^, CI [9.45e*^–^*^3^ – −6.60e*^–^*^3^, *p* ≤ 0.001; PVS volume ß = −1.30e*^–^*^3^, CI [−1.55e*^–^*^3^ – −1.05e*^–^*^3^], *p* ≤ 0.001), as well as between the change in the cognition total composite score and the change in PVS volume (ß = 4.36e*^–^*^3^, CI [1.33e*^–^*^3^ – 7.40e*^–^*^3^], *p* = 0.046) over the trial period. There was a significant association between CSF t-tau and the number of PVS clusters (ß = 2.845, CI [0.630 – 5.06], *p* = 0.036). Additionally, there was a significant relationship between the change in CSF t-tau and the change in the number of PVS (ß = 1.54, CI [0.918 – 2.16], *p* < 0.001) and PVS volume (ß = 13.8, CI [6.37 – 21.1], *p* = 0.003) over the trial period. An association was found between the change in NfL and the change in PVS volume (ß = 1.40, CI [0.272 – 2.52], *p* = 0.045) over time. Within the “non-progressor” group (*n* = 7), there was a significant relationship between the change in the CSF total-tau (t-tau) levels and the change in the PVS burden (PVS cluster (ß = 1.46, CI [0.577 – 2.34], *p* = 0.014; PVS volume ß = 14.6, CI [3.86 – 25.4], *p* = 0.032) over the trial period. Additionally, there was evidence of a significant relationship between the change in NfL levels and the change in the PVS burden over time (PVS cluster ß = 0.296, CI [0.229 – 0.361], *p* ≤ 0.001; PVS volume ß = 3.67, CI [2.42 – 4.92], *p* = 0.002).

**Conclusion:**

Analysis of serial MRI scans 12 months apart in patients with bvFTD demonstrated a relationship between PVS burden and disease severity as measured by the total cognitive composite score and CSF t-tau. Further studies are needed to confirm PVS as a robust marker of neurodegeneration in proteinopathies.

## Introduction

Perivascular spaces (PVS) are cerebrospinal fluid (CSF) filled spaces surrounding the brain’s vasculature. They form part of the brain’s glymphatic clearance system which functions to transport fluid between compartments and remove waste products from the brain (Nedergaard and Goldman, 2016). It is theorized that toxic proteins and metabolites are collected by fluids entering the brain parenchyma and are cleared from the brain *via* PVS (Iliff et al., 2012). As MRI technology has advanced (higher spatial resolution, signal to noise and contrast), enlarged PVS have become more conspicuous. PVS are theorized to become enlarged secondary to poor glymphatic elimination of obstructed waste products, such as in neurodegenerative diseases including late onset Alzheimer’s disease and Frontotemporal Dementias (Weller et al., 2009; Ramirez et al., 2014; Peng et al., 2016). Thus, enlarged PVS visualized on MRI may be a surrogate marker for poor glymphatic clearance in these conditions (Mestre et al., 2017). Many algorithms have been developed for the automated detection of PVS on MRI (Park et al., 2016; Ramirez et al., 2016; Wang et al., 2016; Hou et al., 2017; Ballerini et al., 2018; Boespflug et al., 2018; Dubost et al., 2019; Schwartz et al., 2019; Sepehrband et al., 2019). However, these algorithms typically require multiple volumetric MRI sequences, which are not typically captured in clinical protocols, to accurately detect PVS. An algorithm that was able to detect and measure PVS using a single sequence, such as a volumetric T1 weighted MRI, would vastly improve the clinical utility of these algorithms. This automated algorithm would also have substantial benefits in assessing the utility of PVS as a diagnostic and prognostic biomarker in neurodegenerative diseases.

Behavioural Variant Frontotemporal Dementia (bvFTD) is a neurodegenerative disease characterized by the pathological accumulation of proteins including tau, TAR DNA-binding protein 43 (TDP-43) and fused in sarcoma (FUS) protein amongst others (Hu et al., 2007). Relevant to the pathogenesis of bvFTD, tau has been found to be cleared by the glymphatic system (Iliff et al., 2014; Patel et al., 2019). Therefore, there is potential for PVS to be a biomarker of neurodegeneration in bvFTD. To date, only a single study has investigated PVS in patients with bvFTD, demonstrating enlargement of PVS when compared to healthy controls (Patankar et al., 2005). Longitudinal studies evaluating the changes to PVS number and volume in patients with bvFTD have yet to be completed.

In this study, we performed a longitudinal assessment of PVS burden in patients with bvFTD. We hypothesized that PVS burden is related to other measures of disease severity (cognition, carer burden scale, blood and CSF protein biomarkers) and PVS could be used as a biomarker of disease progression in bvFTD.

## Materials and methods

### Study participants

Patients with possible or probable bvFTD (*n* = 12) were recruited as part of the Phase 1b clinical trial of sodium selenate as a disease modifying treatment for bvFTD (SEL001 study). The main study results are reported elsewhere (Vivash et al., 2021). One was excluded due to missing MRI data, and another was excluded due to poor quality MRI data; the ten remaining participants were included in this study.

The study was performed in accordance with the Declaration of Helsinki and Good Clinical Practice. The study was approved by the local institutional Human Research Ethics Committee (2017.090, Melbourne Health, Melbourne, VIC, Australia) and prospectively registered on the Australian New Zealand Clinical Trials Registry (ACTRN12617001218381). Written informed consent was obtained from the participant or their legally authorized representative (as required by local laws and regulations), and the participant’s carer.

### Procedures and assessments

All participants who completed screening underwent baseline assessments that included MRI, blood and CSF collection (for biomarkers), and an extensive cognitive and behavioral battery. These assessments were all repeated at week 52. Additional safety and other assessments that are outside the scope of the present study were completed up to 9 times throughout the study period and are detailed elsewhere (Vivash et al., 2021).

### Cognition and disease severity

Cognition was assessed using the Neuropsychiatric Unit Cognitive Screening Tool (NUCOG) (Walterfang et al., 2006) and the NIH toolbox cognition battery (Bauer and Zelazo, 2013). The NUCOG is a quick, pen and paper-based test that measures global cognition. Scores range from 0 to 100 with lower scores indicating worsening of cognitive ability. The NUCOG was performed at all study visits. The NIH toolbox cognitive battery is a computerized battery of cognitive tests that also measures global cognition. It produces several scores of individual subtests as well as a global cognitive composite score. The NIH toolbox was performed at baseline, week 26 and week 52.

The Caregiver Burden Scale (CBS) was administered at each visit as a measure for disease severity and progression. Higher scores indicate greater carer burden/disease severity (Zarit et al., 1980).

### Biological fluid collection and analysis

Blood and CSF were collected at Baseline and at Week 52 of treatment. CSF, serum, and plasma were stored at −80°C until use. CSF neurofilament light chain (NfL) was measured using enzyme-linked immunosorbent assays (ELISA). CSF, serum, and plasma total-tau (t-tau) were measured using Single Molecular Array (SIMOA) immunoassays. Assay details can be found elsewhere (Vivash et al., 2021).

### MRI acquisition and pre-processing

T1-weighted (T1w) volumetric MRI sequences were acquired on Siemens 3T Prisma (1 mm^3^ resolution, FOV = 22 × 22 cm, imaging matrix = 256 × 256 × 208). Brain volume was calculated as the sum of the grey matter (GM) and the white matter (WM), extracted from T1w MRIs using SPM12 running in MATLAB (v2018a) (MATLAB, 2018).

### Automatic perivascular spaces detection – Multimodal autoidentification of perivascular spaces

The multimodal autoidentification of perivascular spaces (MAPS) algorithm (Schwartz et al., 2019) is an automated method for segmenting PVSs, based on their intensity and morphology. MAPS measures the PVS burden throughout the entire brain volume including both gray and white matter. MAPS was applied to the cohort’s T1w images, with the following modifications: (1) masking with volumetric FLAIR was removed to enable use with T1w scans alone; (2) an expanded ventricular mask was generated to exclude the ventricles from the WM mask, comprised of the Freesurfer ventricular mask dilated by three voxels multiplied by the FMRIB Software Library (FSL)^1^ CSF mask (Jenkinson et al., 2012); (3) the default sensitivity parameters of MAPS were varied to maximize segmentation performance in this data set (see Optimization of MAPS performance).

### Multimodal autoidentification of perivascular spaces pre-processing

Advanced Normalization Tools (ANTS) (version 3.0)^2^ (Avants et al., 2011) skull stripping was applied to the images for brain extraction. The extracted brain mask was then dilated by two voxels to reinstate any gray matter removed by the stripping. Tissue types were segmented using Longitudinal Freesurfer (version 6.0)^3^ (Dale et al., 1999) into cortical grey matter (GM), deep GM, white matter (WM), ventricles, brainstem, parenchyma and the cerebellum. These outputs were transferred into native space and converted to masks for input into the algorithm.

### Multimodal autoidentification of perivascular spaces processing steps

The first phase of the algorithm, using tools from Analysis of Functional Neuroimages (AFNI) (version 10.0.11),^4^ (Cox, 1996) uses intensity to identify voxels of interest utilizing both a local median score and a difference score. The median score is calculated as the difference between the voxel of interest and the median intensity of its neighboring voxels. The difference score assesses the mean difference in intensity of the voxel of interest and its neighboring voxels.

The second phase of the algorithm, performed in MATLAB (version 2018a; MathWorks, Natick, MA, USA), (MATLAB, 2018) uses size and linearity morphology characteristics to further delineate clusters of voxels that are PVS. A cluster threshold is used to exclude voxels that are not grouped into clusters. The default for this threshold is set at 5 so that only clusters of 5 mL or more are identified as potential PVS. Linearity constraints are placed using principle component analysis. The default linearity threshold is set at 0.8 so that greater than 80% of the variation in location of supra-threshold voxels in a cluster is explained by the first principle component. Clusters with a width (extent perpendicular to first principle component) larger than 15 voxels were excluded.

### Optimization of multimodal autoidentification of perivascular spaces performance

**FIGURE 1:**
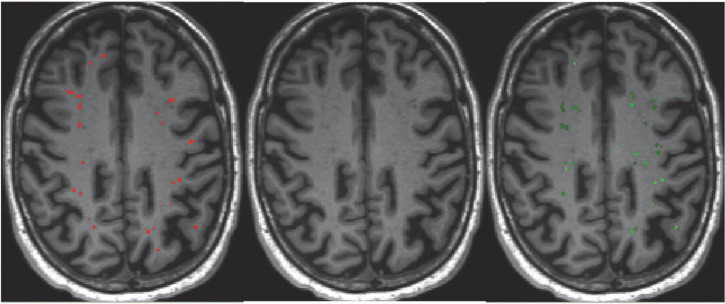
Quantification of perivascular spaces (PVS). PVS burden as shown on a T1-weighted MRI in a patient with bvFTD. **(Left)** Manual segmentation of PVS highlighted in red. **(Right)** Automated segmentation of PVS using the Multimodal Autoidentification of Perivascular Spaces (MAPS) algorithm highlighted in green.

The default median threshold (0.1), difference threshold (0.05), linearity measure (0.8) and cluster threshold (Weller et al., 2009) were iteratively altered to determine the optimal combination of thresholds: difference threshold [0.02, 0.03, 0.04, 0.05, 0.06, 0.07, 0.08, 0.09, 0.1, 0.15], median threshold [0.02, 0.03, 0.04, 0.05, 0.06, 0.07, 0.08, 0.09, 0.1, 0.15], cluster threshold [3, 4, 5, 6, 7, 8,9, 10], and then linearity threshold [0.64, 0.65, 0.66, 0.67, 0.68, 0.69, 0.7, 0.72, 0.75, 0.77, 0.8, 0.82, 0.85, 0.87, 0.9]. The dice score was used to compare the full manual segmentations and MAPS automated segmentations for each combination of thresholds. The combination of thresholds that produced the highest mean dice score was used on the entire dataset of MRI scans (Figure 1).

### Perivascular spaces measurement

The remaining voxels that passed the intensity, cluster, and linearity thresholds were included in the final PVS mask. Visual inspection was required to remove false positives and segment large PVS that were missed. This correction for false negatives were performed for the entire brain volume. The PVS cluster number and volume (number of voxels) were then extracted and adjusted by the white matter volume.

### Validation of multimodal autoidentification of perivascular spaces algorithm

The quality of the segmentation was assessed by comparing the PVS count in a manually segmented axial slice and the corresponding cluster count of the MAPS segmentation in the same slice using the optimal thresholds after visual inspection. Pearson’s correlation between the two counts was used to measure the linear correlation between the manual and automated counts. This was performed on the remaining 16 scans to validate the performance of MAPS.

### Manual rating of perivascular spaces

Manual segmentations were used to validate the performance of the automated segmentations. They were performed on randomly selected T1w MRI scans by a rater blinded to the participant’s clinical information. Full manual segmentations were conducted on four scans with differing PVS burden where PVS were marked in the entire brain volume (Figure 1). Additionally, single axial slice manual segmentations were performed in the centrum semiovale for the remaining 16 scans in line with Wardlaw’s PVS rating scale (Wardlaw et al., 2013).

### Statistical analysis

Wilcoxon signed-rank test was used to assess any differences between baseline and week-52 PVS cluster number and volume measurements. General linear mixed models were computed for each variable of interest, and output data presented as model estimate (ß) and 95% confidence interval. These mixed models adjusted for age, smoking history, blood pressure and time of day in each analysis and the intercept| study number was included as the random co-efficient. Spearman’s correlation was used to determine relationships between baseline PVS measurements and other baseline variables.

Analyses were repeated for the “non-progressor” group alone, participants (*n* = 7) in whom whole brain volume decrease was < 1.8% over the 52 week period on the serial MRI scans. This volume reduction rate represents the 50% reduction rate in the annual brain atrophy percentage observed in patients with bvFTD (3.15%) (Chan et al., 2001). It is hypothesized that those in the “non-progressor” group had a reduced brain atrophy rate potentially due to a therapeutic effect of sodium selenate. Therefore, this sub-group was analyzed to explore any potential associations between slowed disease progression and PVS progression.

Statistical analyses were performed using jamovi (v1.6.15). *P*-values < 0.05 were considered statistically significant.

## Results

### Multimodal autoidentification of perivascular spaces optimization

The optimal parameters found were a difference threshold of 0.05, median threshold of 0.06, cluster threshold of 5, and linearity threshold of 0.68. This achieved a greater performance of the algorithm when compared to the default parameters (mean dice score of 0.211 using default parameters compared to a mean dice score of 0.373 using optimal parameters).

### Multimodal autoidentification of perivascular spaces validation

The correlation between the manual and automated segmentations was not significant using default parameters (*r* = 0.348, *p* = 0.187) or the optimal thresholds after visual inspection (*r* = −0.201, *p* = 0.456). The false positives and negative PVS counts pre- and post-visual inspection can be found in Table 1.

**TABLE 1:** False positive and false negative count pre- and post-visual inspection.

	Pre-visual inspection		Post-visual inspection	
		
	False negative clusters	False positive clusters	False negative clusters	False positive clusters
Subject 1 BL	16	1	16	1
Subject 1 FU	48	3	47	3
Subject 2 BL	0	0	0	0
Subject 2 FU	1	18	4	3
Subject 4 BL	6	23	6	23
Subject 4 FU	8	12	8	12
Subject 5 BL	13	1	13	1
Subject 5 FU	9	0	9	0
Subject 6 BL	10	17	10	17
Subject 6 FU	13	5	18	1
Subject 7 BL	1	46	1	41
Subject 7 FU	4	2	4	1
Subject 8 BL	2	24	2	21
Subject 8 FU	5	34	5	34
Subject 10 BL	5	5	5	3
Subject 10 FU	5	0	5	0
Subject 11 BL	2	40	2	31
Subject 11 FU	6	121	6	80
Subject 12 BL	8	1	8	1
Subject 12 FU	15	81	15	68

The number of false negative and false positive PVS clusters are displayed for each subject with single slice manual segmentations at baseline and at follow-up. PVS, perivascular space; BL, baseline, FU, follow-up.

### Participant demographics

Participant demographics are presented in Table 2. Median age was 62 years (49–72 years) and 8 participants were male. Median disease duration at baseline was 10.5 months (1.5 – 18.5 months).

**TABLE 2:** Baseline patient demographics and descriptive statistics.

	Whole group analysis	Progressor group	Non-progressor group	*P-value*
Participants, n	10	3	7	
Age at entry, years	61.97 (48.66 – 71.6)	58.65 (48.66 – 70.01)	63.69 (55.15 – 71.60)	0.506
**Sex, n**				
Male	8	1	7	0.007**
Female	2	2	0	
Disease duration at entry, months	10.50 (1.50 – 18.55)	2.83 (1.50 – 4.50)	13.78 (6.90 – 18.55)	0.024*
Brain volume change,%	−2.70 (−6.51 – −0.260)	−5.25 (−6.51 – −2.44)	−1.24 (−1.76 – −0.260)	<0.001***
NUCOG BL	73.65 (47.5 – 93)	66.67 (49 – 80)	76.64 (47.5 – 93)	0.315
NUCOG wk 52	65.75 (19 – 97)	40.5 (19 – 73)	76.57 (40.5 – 97)	0.073^
Change in NUCOG score, n	−7.90 (−41.5 – 8.00)	−26.17 (−41.5 – −7.00)	−0.07 (−17 – 8)	0.005**
CSF t-tau BL, ng/mL	152.41 (86.31 – 321.10)	110.77 (93.56 – 127.45)	173.24 (86.31 – 321.10)	0.412
CSF t-tau wk 52, ng/mL	144.00 (75.41 – 216.64)	125.02 (75.41 – 158.29)	153.49 (95.23 – 216.64)	0.548
Change in CSF t-tau, ng/mL	−8.41 (−104.47 – 47.00)	14.26 (−18.16 – 47.00)	−19.74 (−104.47 – 16.93)	0.584
Serum t-tau BL, ng/mL	0.71 (0.02 – 1.87)	0.69 (0.02 – 1.22)	0.72 (0.11 – 1.87)	0.648
Serum t-tau wk 52, ng/mL	0.99 (0.07 – 2.95)	0.60 (0.17 – 1.07)	1.19 (0.07 – 2.95)	0.762
Change in serum t-tau, ng/mL	0.28 (−0.671 – 1.08)	−0.09 (−0.67 – 0.55)	0.47 (−0.44 – 1.08)	0.635
Plasma t-tau BL, ng/mL	3.33 (1.84 – 6.13)	2.53 (1.84 – 3.22)	3.65 (1.93 – 6.13)	0.548
Plasma t-tau wk 52, ng/mL	3.21 (1.36 – 5.30)	1.71 (1.36 – 2.07)	3.81 (2.98 – 5.30)	0.648
Change in plasma t-tau, ng/mL	−0.12 (−3.16 – 3.37)	−0.82 (−1.15 – −0.49)	0.16 (−3.16 – 3.37)	0.855
NfL BL, ng/mL	2189.56 (683.35 – 4211.12)	3340.67 (2454.60 – 4211.12)	1614.00 (683.35 – 2728.84)	0.012*
NfL wk 52, ng/mL	2004.99 (665.59 – 5031.11)	3306.20 (2330.03 – 5031.11)	1354.39 (665.59 – 2241.94)	0.024*
Change in NfL, ng/mL	−184.57 (−1050.34 – 819.99)	−34.47 (−1026.27 – 819.99)	−259.61 (−1050.34 – 63.93)	0.211

Data are presented as mean (range) unless otherwise indicated. Wilcoxon signed-rank test was used to assess age, sex, and disease duration at entry between progressor and non-progressor groups. Mann–Whitney U test for the change in brain volume, cognition, and biomarkers between the progressor and non-progressor groups were conducted, and the p-value is shown in the right column. ***P-value < 0.001, **p-value < 0.01, *p-value < 0.05, ^*p*-value < 0.1.

### Perivascular spaces burden

The PVS measurements between the baseline and follow up are shown in Table 3. There was no significant change to PVS cluster number (ß = −3.27, CI [−7.80 – 1.27], *p* = 0.267) or PVS volume (ß = −36.8, CI [−84.9 – 11.3], *p* = 0.171) over the trial period.

**TABLE 3:** Perivascular space (PVS) measurements at baseline and follow-up.

	PVS cluster number			PVS volume		
		
	Whole group (*n* = 10)	Progressor group (*n* = 3)	Non-progressor group (*n* = 7)	Whole group (*n* = 10)	Progressor group (*n* = 3)	Non-progressor group (*n* = 7)
Baseline	213.8 (64 – 508)	92 (64 – 121)	266 (90 – 508)	2523.6 (632 – 4603)	1044.3 (632 – 1510)	3157.6 (1248 – 4603)
Follow-up	144.9 (83 – 318)	124.7 (83 – 204)	153.6 (91 – 318)	1660.3 (807 – 3858)	1094 (807 – 1628)	1903 (812 – 3858)

Data are presented as mean and range.

There was no significant relationship between age and PVS cluster number (ß = −0.370, CI [−22.1 – 21.3], *p* = 0.974) or PVS volume (ß = −56.0, CI [−331 – 219], *p* = 0.702). There was no evidence of a relationship between disease duration and PVS cluster number (ß = −0.702, CI [−16.4 – 15.0], *p* = 0.932), or PVS volume (ß = −38.7, CI [−218 – 140], *p* = 0.678).

#### Cognition and disease severity

**FIGURE 2:**
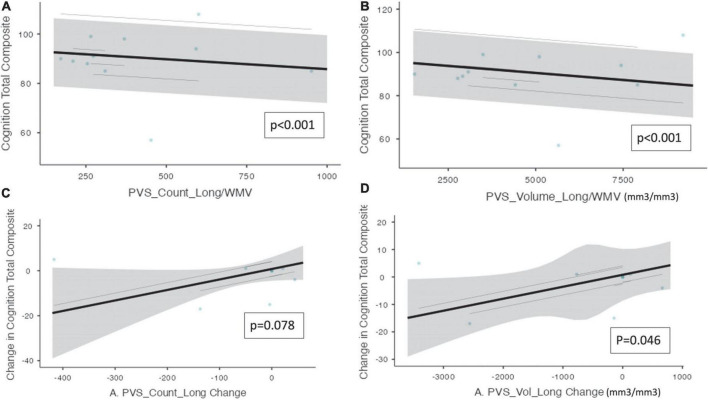
Perivascular spaces measurements and NIH cognition total composite score. Demonstrates a significant relationship between PVS measurements and cognition as measured by the NIH cognition total composite score (*n* = 10). The PVS cluster number and PVS volume were both adjusted by white matter volume. **(A)** PVS cluster number and cognition total composite score. **(B)** PVS volume and cognition total composite score. **(C)** The change in PVS cluster numbers and the change in cognition total composite scores longitudinally. **(D)** The change in PVS volume and the change in cognition total composite scores longitudinally. Individual participants are depicted as blue dots. The gray band represents the 95% confidence interval, and the black line represents the estimated mean.

Mixed model analysis for PVS burden and NUCOG, cognition total composite scores, and CBS are presented in Table 4. There was a significant relationship between cognition total composite scores and the number of PVS (ß = −0.802e*^–^*^3^, CI [9.45e*^–^*^3^ – −6.60e*^–^*^3^, *p* ≤ 0.001) and the PVS volume (ß = −1.30e*^–^*^3^, CI [−1.55e*^–^*^3^ – −1.05e*^–^*^3^], *p* ≤ 0.001, Figure 2). There was a significant relationship between the change in cognition total composite score and the change in PVS volume (ß = 4.36e*^–^*^3^, CI [1.33e*^–^*^3^ – 7.40e*^–^*^3^], *p* = 0.046), and a trend toward a significant relationship between the change in cognition total composite score and the change in the number of PVS (ß = 0.047, CI [0.005 – 0.088], *p* = 0.078) over the trial period.

**TABLE 4:** Perivascular space measurements and cognition and disease severity.

	PVS cluster number			PVS volume		
		
	ß Estimate	Confidence Interval	*P-value*	ß Estimate	Confidence interval	*P-value*
NUCOG	−0.016	−0.046 – 0.014	0.323	−1.21e^–3^	−3.96e^–3^ – 1.53e^–3^	0.402
Cognitive Total Composite Score	−0.802e^–3^	9.45e^–3^ – −6.60e^–3^	< 0.001***	−1.30e^–3^	−1.55e^–3^ – −1.05e^–3^	<0.001***
CBS	5.41e^–4^	−0.021 – 0.022	0.962	1.24e^–4^	−1.95e^–3^ – 2.20e^–3^	0.909

	**Change in PVS Cluster Number**			**Change in PVS Volume**		
		
	**ß Estimate**	**Confidence Interval**	***P-value***	**ß Estimate**	**Confidence Interval**	***P-value***

Change in NUCOG	−0.028	−0.071 – 0.015	0.224	−2.09e^–3^	−6.48e^–3^ – 2.30e^–3^	0.373
Change in Cognition Total Composite	0.047	0.005 – 0.088	0.078^	4.36e^–3^	1.33e^–3^ – 7.40e^–3^	0.046*
Change in CBS	−0.015	−0.053 – 0.024	0.474	7.37e^–5^	−3.81e^–3^ – 3.95e^–3^	0.971

	**PVS Cluster Number**			**PVS Volume**		
		
	**ß Estimate**	**Confidence Interval**	***P-value***	**ß Estimate**	**Confidence Interval**	***P-value***

NUCOG	−0.016	−0.046 – 0.014	0.323	−1.21e^–3^	−3.96e^–3^ – 1.53e^–3^	0.402
Cognitive Total Composite Score	−0.802e^–3^	9.45e^–3^ – −6.60e^–3^	<0.001***	−1.30e^–3^	−1.55e^–3^ – −1.05e^–3^	< 0.001***
CBS	5.41e^–4^	−0.021 – 0.022	0.962	1.24e^–4^	−1.95e^–3^ – 2.20e^–3^	0.909

	**Change in PVS Cluster Number**			**Change in PVS Volume**		
		
	**ß Estimate**	**Confidence Interval**	***P-value***	**ß Estimate**	**Confidence Interval**	***P-value***

Change in NUCOG	−0.028	−0.071 – 0.015	0.224	−2.09e^–3^	−6.48e^–3^ – 2.30e^–3^	0.373
Change in Cognition Total Composite	0.047	0.005 – 0.088	0.078^	4.36e^–3^	1.33e^–3^ – 7.40e^–3^	0.046*
Change in CBS	−0.015	−0.053 – 0.024	0.474	7.37e^–5^	−3.81e^–3^ – 3.95e^–3^	0.971

The β estimate, 95% confidence interval, and *p*-values for the relationship between PVS measurements and cognition, and disease severity are shown. ***P-value < 0.001, **p-value < 0.01, *p-value < 0.05, ^*p*-value < 0.1.

#### Protein biomarkers

**FIGURE 3:**
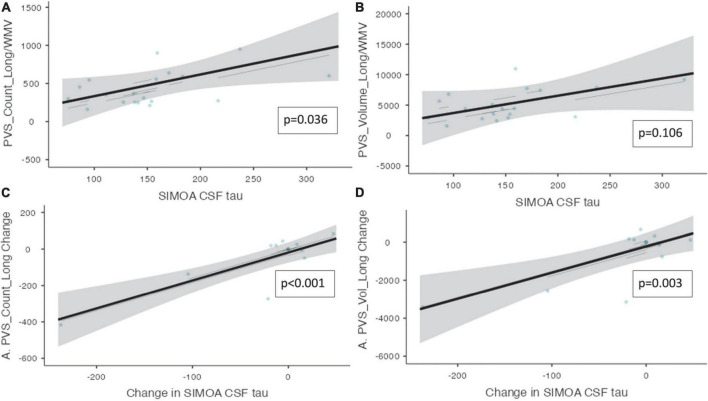
Perivascular spaces measurements and CSF t-tau and NfL. Demonstrates the relationship between PVS measurements and CSF t-tau is shown (*n* = 10). **(A)** The number of PVS clusters and the CSF t-tau measurements. **(B)** The volume of PVS and the CSF t-tau measurements. **(C)** The change in PVS cluster number and change in CSF t-tau longitudinally. **(D)** The change in PVS volume and the change in CSF t-tau longitudinally. Individual participants are depicted as blue dots. The gray band represents the 95% confidence interval, and the black line represents the estimated mean.

Perivascular spaces burden and biofluid biomarkers are shown in Table 5. There was a significant association between CSF t-tau and the number of PVS clusters (ß = 2.845, CI [0.630 – 5.06], *p* = 0.036). Additionally, there was a significant relationship between the change in CSF t-tau and the change in the PVS cluster number (ß = 1.54, CI [0.918 – 2.16], *p* ≤ 0.001) and PVS volume (ß = 13.8, CI [6.37 – 21.1], *p* = 0.003, Figure 3) over the trial period.

**TABLE 5:** Perivascular space measurements and protein biomarkers.

	PVS cluster number			PVS volume		
		
	ß Estimate	Confidence Interval	*P-value*	ß Estimate	Confidence Interval	*P-value*
CSF t-tau	2.845	0.630 – 5.06	0.036*	28.4	−2.28 – 59.0	0.106
Serum t-tau	−202	−560 – 154	0.288	−2179	−5759 – 1401	0.286
Plasma t-tau	122	−0.427 – 245	0.079^	1408	221 – 2594	0.052^
NfL	−0.074	−0.218 – 0.070	0.332	−0.916	−2.66 – 0.825	0.328

	**Change in PVS cluster number**			**Change in PVS volume**		
		
	**ß Estimate**	**Confidence Interval**	***P-value***	**ß Estimate**	**Confidence Interval**	***P-value***

Change in CSF t-tau	1.54	0.918 – 2.16	< 0.001***	13.8	6.37 – 21.1	0.003**
Change in Serum t-tau	−153	−289 – −17.5	0.051^	−1387	−2723 – −50.9	0.076^
Change in Plasma t-tau	25.2	−13.7 – 64.1	0.227	239	−164 – 642	0.266
Change in NfL	0.101	5.93e^–3^ – 0.196	0.085^	1.40	0.272 – 2.52	0.045*

The β estimate, 95% confidence interval, and *p*-values for the relationship between PVS measurements and protein biomarkers are shown. ***P-value < 0.001, **p-value < 0.01, *p-value < 0.05, ^*p*-value < 0.1.

There was a trend toward significant relationships between *plasma* t-tau levels and the PVS count (ß = 122, CI [−0.427 – 245], *p* = 0.079) and PVS volume (ß = 1408, CI [221 – 2594], *p* = 0.052). Additionally, there was a trend toward a significant relationship between the change in *serum* t-tau and the change in the number of PVS (ß–153, CI [−289 – −17.5], *p* = 0.051) and the change in PVS volume (ß = −1387, CI [−2723 – −50.9], *p* = 0.076) over 52-week treatment period.

**FIGURE 4:**
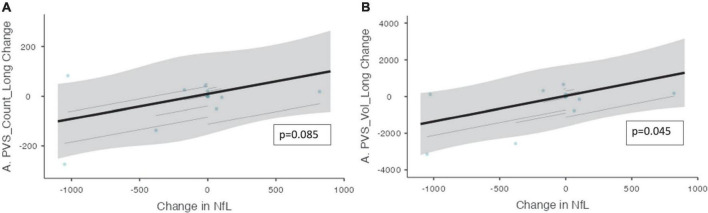
The change in PVS measurements and the change in CSF NfL over the trial period. The relationship between PVS measurements and CSF NfL (*n* = 10) over the trial period is shown. **(A)** The change in the number of PVS clusters and the change in CSF NfL longitudinally. **(B)** The change in PVS volume and the change in CSF NfL longitudinally. Individual participants are depicted as blue dots. The gray band represents the 95% confidence interval, and the thick black line represents the estimated mean.

An association was found between the change in NfL and the change in PVS volume (ß = 1.40, CI [0.272 – 2.52], *p* = 0.045, Figure 4). Moreover, over the trial period, there was also a trend toward a significant relationship between the change in NfL and the change in the PVS count (ß = 0.101, CI [5.93e*^–^*^3^ – 0.196], *p* = 0.085).

### Non-progressor group analysis

There was a significant difference in the disease duration at baseline between the progressor and non-progressor groups (2.83 vs. 10.50 months, *p* = 0.024). There was a clear distinction between progressor and non-progressor groups based on brain volume changes and cognitive scores, as shown in Table 2. Exploratory analysis was conducted on the non-progressor group to assess any associations of PVS to different variables.

#### Time

Within the non-progressor group, there was a significant change to the number of PVS clusters (ß = −7.461, CI [−13.1 – −1.84], *p* = 0.035) and the PVS volume (ß = −72.6, CI [−130 – −15.6], *p* = 0.0.041) over the trial period.

#### Cognition and disease severity

Mixed model analysis for PVS burden and NUCOG, cognition total composite scores, and CBS are presented in Table 6. There was a significant relationship between the cognition total composite scores and the number of PVS (ß = −8.07e*^–^*^3,^ CI [−9.76e*^–^*^3^ – −6.38e*^–^*^3^], *p* = 0.003) and the PVS volume (ß = −1.31e*^–^*^3^, CI [−1.61e*^–^*^3^ – −1.02e*^–^*^3^], *p* = 0.003).

**TABLE 6:** Perivascular space measurements and cognition, and disease severity within the non-progressor group.

	PVS cluster number			PVS volume		
		
	ß Estimate	Confidence Interval	*P-value*	ß Estimate	Confidence Interval	*P-value*
NUCOG	−8.95e^–3^	−0.030 – 0.012	0.448	−1.06e^–3^	−3.19e^–3^ – 1.08e^–3^	0.381
Cognition Total Composite	−8.07e^–3^	−9.76e^–3^ – −6.38e^–3^	0.003**	−1.31e^–3^	−1.61e^–3^ – −1.02e^–3^	0.003**
CBS	−4.34e^–3^	−9.81e^–3^ – 1.12e^–3^	0.214	−3.65e^–4^	−1.00e^–3^ – 2.96e^–4^	0.336

	**Change in PVS Cluster Number**			**Change in PVS Volume**		
		
	**ß Estimate**	**Confidence Interval**	***P-value***	**ß Estimate**	**Confidence Interval**	***P-value***

Change in NUCOG	−0.015	−0.048 – 0.018	0.395	−1.00e^–3^	−4.07e^–3^ – 2.07e^–3^	0.544
Change in Cognition Total Composite	0.017	−0.022 – 0.055	0.481	3.87e^–3^	1.34e^–3^ – 6.39e^–3^	0.184
Change in CBS	−9.18e^–3^	−0.021 – 0.002	0.213	−6.95e^–4^	−2.04e^–3^ – 6.47e^–4^	0.379

The β estimate, 95% confidence interval, and p-values for the relationship between PVS measurements and cognition, and disease severity within the non-progressor group are shown.

***P-value < 0.001, **p-value < 0.01, *p-value < 0.05, ^*p*-value < 0.1.

#### Biomarkers

**FIGURE 5:**
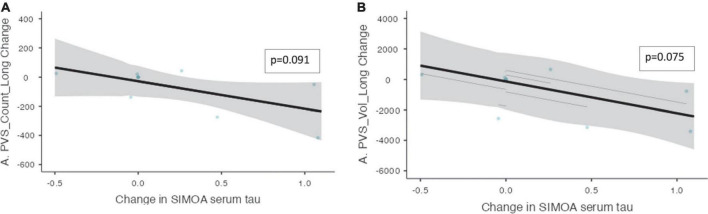
The change in PVS measurements and the change in serum t-tau over the trial period within the non-progressor group. The relationships between PVS measurements and serum t-tau levels within the non-progressor group are shown over the trial period (*n* = 7). **(A)** The change in PVS cluster numbers and the change in serum t-tau longitudinally. **(B)** The change in PVS volume and the change in serum t-tau longitudinally. Individual participants are depicted as blue dots. The gray band represents the 95% confidence interval, and the thick black line represents the estimated mean.

Mixed model analysis for the PVS burden and biomarkers are presented in Table 7. The change in CSF t-tau levels was associated with the change in the number of PVS (ß = 1.46, CI [0.577 – 2.34], *p* = 0.014) and the volume of PVS (ß = 14.6, CI [3.86 – 25.4], *p* = 0.032) over the trial period. There was a trend toward a significant relationship between the change in the *serum* t-tau levels and the change in the number of PVS (ß = −188, CI [−376 – −0.069], *p* = 0.091) and the change in the PVS volume (ß = −2084, CI [−3860 – −307], *p* = 0.075, Figure 5) over the 52 weeks. An association was found between the change in NfL levels and the change in number of PVS (ß = 0.296, CI [0.229 – 0.361], *p* ≤ 0.001) and the PVS volume (ß = 3.67, CI [2.42 – 4.92], *p* = 0.002) over the trial period.

**TABLE 7:** Perivascular space measurements and biomarkers within the non-progressor group.

	PVS cluster number			PVS volume		
		
	ß Estimate	Confidence Interval	*P-value*	ß Estimate	Confidence Interval	*P-value*
CSF t-tau	2.73	−0.205 – 5.66	0.155	18.1	−15.9 – 52.0	0.373
Serum t-tau	−286	−761 – 189	0.282	−4590	−9150 – −30.1	0.113
Plasma t-tau	176	−1.51 – 353	0.110	1662	5.14 – 3319	0.106
NfL	0.038	−0.421 – 0.496	0.880	−1.66	−5.34 – 2.03	0.412

	**Change in PVS Cluster Number**			**Change in PVS Volume**		
		
	**ß Estimate**	**Confidence Interval**	***P-value***	**ß Estimate**	**Confidence Interval**	***P-value***

Change in CSF t-tau	1.46	0.577 – 2.34	0.014*	14.6	3.86 – 25.4	0.032*
Change in Serum t-tau	−188	−376 – -0.069	0.091^	−2084	−3860 – -307	0.075^
Change in Plasma t-tau	22.9	−33.3 – 79.1	0.451	261	−349 – 871	0.430
Change in NfL	0.296	0.229 – 0.361	<0.001***	3.67	2.42 – 4.92	0.002**

The β estimate, 95% confidence interval, and *p*-values for the relationship between PVS measurements and various biomarkers within the non-progressor group are shown. ***P-value < 0.001, **p-value < 0.01, *p-value < 0.05, ^*p*-value < 0.1.

## Discussion

This study evaluated the relationship between changes in PVS burden on serial MRIs and biomarkers for neurodegeneration over 12 months in patients with bvFTD. PVS burden was associated with cognition, CSF t-tau levels, and CSF NfL levels; and in patients who did not show disease progression, PVS burden was also associated with cognition, CSF t-tau levels, and CSF NfL levels.

### Perivascular spaces as a marker of neurodegeneration

Neither PVS cluster number nor volume changed over time in this patient cohort. Previous longitudinal studies in cerebral small vessel disease and Parkinson’s disease have demonstrated increased PVS burden over time (Dubost et al., 2019; Shibata et al., 2019; Shen et al., 2021), however, this is the first study to investigate PVS longitudinally in bvFTD. The lack of progression in PVS burden seen in this cohort could suggest that is not a good marker of disease progression in this condition, or alternatively it could potentially be due to a therapeutic effect of sodium selenate slowing disease progression. The mechanism of action of sodium selenate in reducing neurodegeneration is by activation of PP2A and subsequent reduction of hyperphosphorylated tau in the brain. It is hypothesized that proteins such as tau and fibrin obstruct and enlarge the PVS, treatment with sodium selenate reduces tau and causes enlarged PVS to shrink, reducing both number and volume. Analysis of the “non-progressor” group, in whom cognitive and behavioral decline and brain atrophy rates were minimal, demonstrated a reduction in PVS cluster number and volume over the trial period. Malpas et al. found less neurodegeneration in the white matter for patients with Alzheimer’s disease treated with sodium selenate compared to those on placebo (Malpas et al., 2016). However, this is the first cohort of patients with bvFTD in which the disease-modifying effects of sodium selenate have been tested (Vivash et al., 2020, 2021).

### Relationship between perivascular spaces and other markers of disease severity

There was a significant positive relationship between both the number and PVS and the PVS volume with cognition measured by the NIH toolbox, suggesting poorer cognitive function in patients with lesser PVS burden. This is in contrast to other studies which found associations between poorer cognitive function and higher PVS burden (MacLullich et al., 2004; Park et al., 2019; Passiak et al., 2019; Jie et al., 2020; Paradise et al., 2021). In particular, they found an association between PVS measured by manual rating scales and executive functioning in cohorts of patients with mild cognitive impairment or Alzheimer’s disease. To date, there is no literature evaluating the relationship between cognition and PVS burden in patients with bvFTD. Similarly, no other studies have reported the use of the NIH toolbox in patients with bvFTD. Larger studies are needed to confirm the use of the NIH toolbox in bvFTD as well as further investigate the relationship with PVS.

There were significant positive relationships between the change in the number of PVS with the change in t-tau levels. This is in agreement with previous studies in Alzheimer’s and Parkinson’s disease (Fang et al., 2020; Vilor-Tejedor et al., 2021), thus our findings add to the body of literature that increased PVS burden is a marker of neurodegeneration. This was further supported by the relationship between PVS burden and NfL levels, whereby greater change in PVS number correlated with greater change in NfL levels. This finding is also supported by the literature, with higher CSF NfL levels correlating with enlarged PVS in amyloid-positive cognitively unimpaired patients (Vilor-Tejedor et al., 2021).

### The effect of sodium selenate on t-tau and perivascular spaces

The exploratory analysis of the non-progressor group demonstrated a trend toward a negative relationship between change in serum t-tau levels and change in the number of PVS clusters. This is in line with the proposed mechanism of action of sodium selenate, whereby increased PP2A activity in the CNS would lead to clearance of tau from the brain, and increased levels of t-tau in the periphery. Theoretically, if there are fewer protein aggregates clogging the glymphatic system in the brain, the flow of CSF through the glymphatic system improves, and PVS reduces in number and volume. Thus, fewer enlarged PVS clusters can be detected on MRI. The significance of this change in t-tau levels in the periphery is not well established. To date, there has only been one study evaluating peripheral measurements of t-tau in frontotemporal lobar degeneration longitudinally. Illan-Gala et al. found no significant changes to plasma t-tau levels over the study period (Illán-Gala et al., 2021). Overall, our findings show that a decrease in PVS cluster number was associated with increase tau levels in the periphery, which may be an effect of sodium selenate treatment mediated by improved glymphatic clearance. Further studies to demonstrate the relationship between PVS and accumulation of pathogenic proteins are needed, as is a randomized controlled trial to further investigate the use of sodium selenate as a treatment for bvFTD (Vivash et al., 2020).

### Limitations

There are a number of limitations associated with this study. Firstly, the cohort size is small and although these preliminary results are encouraging, are not adequate to draw definitive conclusions from the results. Additionally, due to the proposed mechanism of sodium selenate, these findings may not translate to the natural progression of bvFTD. Moreover, it was difficult to determine the underlying mechanism driving the changes to PVS size and number. It is uncertain whether the changes observed were due to changes to glymphatic function, measurement variability, another disease mechanism, or the quality of the individual scans. Given the variable image quality (due to motion artifacts) manual visual inspection and validation was important to maximize the validity of the data created by the algorithm.

There was also a gender bias, with eight out of the ten participants being male. There are no significant gender differences in clinical presentations in bvFTD. However, sex differences in PVS burden have been described and thus may contribute to our results (Kuribara et al., 2017; Piantino et al., 2020). This gender bias was mitigated by adjusting the PVS measurements by brain volume. However, future studies are encouraged to have a more equal balance of male and female subjects to mitigate any gender differences further. The CBS is a subjective measurement of disease severity. However, it is a well validated measure of disease severity. Additionally, our results for CBS were aligned with objective measures of disease severity including CSF t-tau and NfL. Future studies require a randomized trial to mitigate any bias from this subjective measure.

There was poor correlation between the manual counts and automated segmentations likely due to low quality images and the presence of motion artifacts. The patient cohort, due to the cognitive effects of their disease, can struggle to lie still whilst inside the MRI scanner. The scans from this dataset were of similar quality typically seen in clinical practice, with low contrast to noise ratios (CNR). A higher CNR represents a greater distinction in intensity between the dark intensity of the CSF-filled PVS and the light intensity of the surrounding WM. Therefore, higher CNR allows for PVS to be more easily identified. MAPS performs much more favorably when used on higher quality datasets with higher CNR [10, Major, Sinclair et al. (under review)] Therefore, future research should optimize PVS segmentation for scan qualities usually taken in clinical practice.

## Conclusion

This is the first study to assess PVS on MRIs acquired longitudinally in a cohort of bvFTD patients. We found a trend toward a significant positive association between the change in CBS and the change in PVS volume longitudinally over 12 months, as well as between the measures of CSF t-tau and PVS burden longitudinally. Further studies are warranted to establish the potential of PVS as a marker of disease progression and neurodegeneration in proteinopathies.

## Data availability statement

Data will be available upon reasonable request to the approving ethics committee. Requests to access the datasets should be directed to LV, lucy.vivash@monash.edu.

## Ethics statement

The studies involving human participants were reviewed and approved by the Melbourne Health Human Research Ethics Committee (ref 2017.090). The patients/participants provided their written informed consent to participate in this study.

## Author contributions

BS, DS, LS, TO’B, ML, and LV conceived and designed the research, edited, and revised the manuscript. JM analyzed the data, drafted the manuscript, and prepared the figures. All authors interpreted the results and approved the final manuscript.

## Footnotes

1
http://fsl.fmrib.ox.ac.uk/fsl/fslwiki/FSLVBM

2
http://stnava.github.io/ANTs/

3
http://surfer.nmr.mgh.harvard.edu/

4
http://afni.nimh.nih.gov/afni
